# Correspondence

**Published:** 1885-01

**Authors:** 


### CORRESPONDENCE.

EDITOR DENTAL REGISTER:

*Dear Sir*—Believing a short sketch of some things essentially English in the practice of dental surgery might prove interesting to your readers, will proceed to give you the benefit of my observations during my short stay here. One thing I have noticed in connection with the operative branch is the studious avoidance of any means whereby a patient's mouth may be put in a healthy and clean condition prior to any operation upon the teeth; such neglect entailing upon the patient the eventual loss of natural organs, that, had a correct course been followed out, the results would have redounded to the credit of the dentist to say nothing of the practical benefit to the patient, who, instead of becoming a subject of loathing and disgust, would be happy, and pleasant to come in contact with.

I have seen patients who have been under the treatment of their dentists for years, visiting them two or three times a year during that time, with their mouths in the most unsanitary condition possible—incrustations of tartar extending deeply beneath the gums and extending above to such an extent as to almost entirely cover the teeth, the condition of the gums in such cases can better be imagined than described. Of course such a state of affairs is reflex in its action, producing, on the part of the patient, an indifference to the hygiene of the oral cavity that, in in some cases, is appalling.

We can't wonder at the desire of the majority of dentists here being content with inserting a plastic or amalgam filling, and seeing the door close behind the patient.

The dentist, too, in continually coming in contact with such a depraved state of affairs, maps out an easy course of practice which, together with the indifferent means of instruction and almost total ignorance of principles, slowly but surely drops into a rut from which death alone releases him; such termination being the only successful event in a career that has been principally devoted to the profound study of—fees.

I think it is the abuse of gold here that has made this a country of "pot metal" and "lath and plaster" dentists. I have heard some persons call *filling* a tooth, "stuffing a tooth," such appellation being the result of the impression conveyed to the mind of the patient by the course of practice pursued by the dentist.

The gold mostly used here is soft gold, the manipulation of cohesive being but little understood, besides entailing a thorough manipulation to produce perfect results that grossly conflicts with the ideas of most practitioners here, who consider the easiest applied filling the best, hence the preference of soft gold over cohesive.

I have seen some soft foil fillings that have stood well for years, but as a rule they were in cavities that were easily accessible, and requiring but little skill in their perfecting; but where approximal cavities needed fiilling the material was almost invariably amalgam, Hill's stopping, or some of the cements.

Have seen but very few attempts at contour work, which has invariably been performed by a few London dentists.

In regard to the treatment of exposed pulps, devitalized teeth, etc., they know practically nothing. Should a pulp die, they, in all probability, will erect an amalgam monument over it, sacred to the memory of the dear departed—i. e. the pulp—the tooth departs later on—when the effects of such treatment goes to work and raises a monument, in the shape of an abscess, to the memory of the incompetency of the dentist, who, when applied to for relief, either advises waiting, or extracts the tooth there and then, and with a nonchalance that would cause the veriest stoic to turn green with envy, pockets the fee for the extraction with as much gusto as he did the fee for the--filling (?).

I believe there are only five or six English dentists that conscientiously go to work to save devitalized teeth. They are meeting with fair success. Nearly or quite all of the American dentists here have brought their modes of practice with them, but their efforts, I am afraid, are not appreciated at their true value, for the public are not imbued with the spirit of rapid advancement that the people of the United States are noted for, and while appreciating to quite an extent the advance of their trans-atlantic cousins in dental matters, they prefer to have their operations performed in good *old* English style, because their fathers did. What a grand thing filial affection is!

More anon, "78."

LONDON, ENG., Dec. 5, 1884.

---

TO THE EDITOR OF DENTAL REGISTER:

*Dear Sir:*—On page 550 of the REGISTER Dr. Robinson calls attention to the use of a mixture of equal parts of carbolic acid and caustic potash as an application to sensitive dentine. He states that the remedy works by coagulation.

When carbolic acid and caustic potash are mixed they form carbolate of potash, which has not as much power to coagulate albumen as the pure carbolic acid, and is not as efficient in practical use in the teeth.

Carbolic acid is an old remedy for this purpose, and is only occasionally efficient, why, therefore, make it less efficient by converting it into a less active substance?

Substances which coagulate albumen are certainly of some value for diminishing the sensibility of teeth, but it is better to combine them with other substances which have the power of absorbing water from the tissues, and other preparations which are sedatives or narcotics.

Caustic potash absorbs water, and if present in excess in Dr. Robinson's mixture has value, but not enough to compensate for the injury which has resulted through its combination with the carbolic acid.

Absolute alcohol, or glycerine is of more value for this purpose *i.e.*, mixing with carbolic acid, because while they take up water they do not form new chemical compounds with the phenol.

A simple mixture of the three classes of substances to which I have called attention, is as follows:

Glycerine with as much carbolic acid as it will take up. To this add as much chloral, or bromal, or butylchloral as the mixture will hold. To this add one-third of its weight of chloroform.

I have given considerable attention to experiments and many chemicals for diminishing the sensibility of teeth and will at some future time send a list of those which I have tried, as this may be of some value by preventing others from buying those which I have found useless for the purpose.

I ought to mention that the chloral, butylchloral, or bromal should be anhydrous. Yours sincerely,

William Herbert Rollins.

28 Chestnut St., Boston, Nov. 26.

